# Quantification of isocyanates and amines in polyurethane foams and coated products by liquid chromatography–tandem mass spectrometry

**DOI:** 10.1002/fsn3.88

**Published:** 2014-01-23

**Authors:** Motoh Mutsuga, Miku Yamaguchi, Yoko Kawamura

**Affiliations:** National Institute of Health Sciences1-18-1, Kamiyoga, Setagaya-ku, Tokyo, 158-8501, Japan

**Keywords:** Amine, isocyanate, liquid chromatography–tandem mass spectrometry, polyurethane

## Abstract

An analytical method for the identification and quantification of 10 different isocyanates and 11 different amines in polyurethane (PUR) foam and PUR-coated products was developed and optimized. Isocyanates were extracted and derivatized with di-*n*-butylamine, while amines were extracted with methanol. Quantification was subsequently performed by liquid chromatography–tandem mass spectrometry. Using this methodology, residual levels of isocyanates and amines in commercial PUR products were quantified. Although the recoveries of certain isocyanates and amines were low, the main compounds used as monomers in the production of PUR products, and their decomposition species, were clearly identified at quantifiable levels. 2,4-and 2,6-toluenediisocyanate were detected in most PUR foam samples and a pastry bag in the range of 0.02–0.92 mg/kg, with their decomposition compounds, 2,4-and 2,6-toluenediamine, detected in all PUR foam samples in the range of 9.5–59 mg/kg. PUR-coated gloves are manufactured using 4,4′-methylenebisphenyl diisocyanate as the main raw material, and a large amount of this compound, in addition to 4,4′-methylenedianiline and dicyclohexylmethane-4,4′-diamine were found in these samples.

## Introduction

Polyurethane (PUR) is obtained from the chemical reaction between diisocyanates and polyols. The reaction of isocyanates with water results in the formation of amines and carbon dioxide, which then acts as a blowing agent. Flexible PUR foam is used as a cushioning for fruits, absorbent pad for fish and meats, and in kitchen sponges. In addition, PUR-coated products such as gloves and pastry bags are used in applications involving contact with food.

PUR is commonly made from 2,4-or 2,6-toluene diisocyanate (2,4-or 2,6-TDI) and sometimes from 4,4′-methylenebisphenyl diisocyanate (4,4′-MDI) (Takayanagi 2000). Therefore, most PUR products also contain 2,4-or 2,6-toluenediamine (2,4-or 2,6-TDA) or 4,4′-methylenedianiline (4,4′-MDA) (Colleen et al. 1985; Inoue et al. 1985; Hull et al. 1989; Lawson et al. 1996). Several isocyanates and amines such as TDA and MDA are listed as possible human carcinogens (WHO/IPCS 1987; ILO and WHO 2000). In Regulation (EU) No 10/2011 (EC 2011), 14 aliphatic and aromatic isocyanates are listed as monomers and other starting substances that are authorized for the manufacture of plastic materials and articles that are intended to come into contact with foodstuffs. Their residual limit in the finished articles is 1.0 mg/kg, expressed as the isocyanate function (NCO) (EC 2011). According to this regulation, primary aromatic amines should not be released into foods or food simulants in detectable quantities. For the purpose of analysis, the detection limit for amines is set at 0.01 mg/kg foods or food simulants. The Food and Drug Administration (FDA) in the USA regulates PUR resins in indirect food additives in 21 Code of Federal Regulation (CFR) 177.1680 as only to be used in dry solid foods with a surface containing no free fat or oil (US FDA 2009).

Many methods for identifying and investigating isocyanates or amines in PUR products have been published (Colleen et al. 1985; Inoue et al. 1985; Hull et al. 1989; Damant et al. 1995; Lawson et al. 1996; Marand et al. 2004; Karlsson et al. 2005; ISO 2006); however, levels of individual residual isocyanates and amines in the same product have not been reported to date.

For isocyanate and amine analysis, liquid chromatography–tandem mass spectrometry (LC/MS/MS) detection can allow for highly sensitive and selective identification. In this study, we have optimized this analytical method for 10 isocyanates and 11 amines in PUR products. We have then quantitatively evaluated the residual levels of isocyanates and amines in commercial flexible PUR foams and PUR-coated products. The relationship between the levels of the different components is also discussed.

## Materials and Methods

### Sample materials

Nine PUR foam products and three PUR-coated products were purchased from Japanese retail outlets. The PUR foam products consisted of three cushionings for fruits and six kitchen sponges. The PUR-coated products consisted of two types of nylon fiber glove that were coated with PUR on the palm side, and one polyester pastry bag (otherwise known as a piping bag or forcing bag) that was coated with PUR on one side. The kitchen sponges consisted of two or three parts, which increased the sample number to 12.

### Reagents

The isocyanate and amine standards used in this study are listed in Table 1. Stock solutions of each amine (1000 *μ*g/mL) were prepared in acetone (poly chlorinated biphenyl analytical grade, Sigma–Aldrich, Tokyo, Japan), and those of each isocyanate (1000 *μ*g/mL) were prepared in toluene (Sigma–Aldrich).

**Table 1:** Isocyanate and amine standards used in this study.

Group	Code	Compound	CAS no.	Purity, %^1^	Retention time, min	Cone voltage, *V*	Collision energy, eV	Quantitative ion, *m/z*	Qualifying ion, *m/z*
Isocyanate	PI	Phenyl isocyanate	103-71-9	>98^a^	1.85	35	16	249.1→130.1	249.1→120.1
	CHI	Cyclohexyl isocyanate	3173-53-3	>98^a^	2.17	35	18	255.1→130.1	255.1→87.1
	HDI	Hexamethylene diisocyanate	822-06-0	>98^a^	2.96	40	30	427.3→130.1	427.3→298.2
	2,6-TDI	2,6-Toluene diisocyanate	91-08-7	>95^a^	3.13	55	26	433.3→130.1	433.3→304.2
	2,4-TDI	2,4-Toluene diisocyanate	584-84-9	>99^a^	3.37	55	26	433.3→130.1	433.3→304.2
	IPDI^2^	Isophorone diisocyanate	4098-71-9	>98^a^	4.08, 5.15	45	28	481.5→130.1	481.5→3522
	TMDI^3^	Trimethylhexamethylene-l,6-diisocyanate	28679-16-5	>97^a^	4.27, 4.49	45	30	469.5→130.1	469.5→340.2
	4,4′-MDI	4,4′-Methylenebisphenyl diisocyanate	101-68-8	>98^a^	4.37	55	28	509.4→130.1	509.4→380.3
	H12MDI^4^	Dicyclohexylmethane-4,4′-diisocyanate	5124-30-1	>90^a^	5.13	45	30	521.4→130.1	521.4→3923
	ODI	Octadecyl isocyanate	112-96-9	>75^a^	11.33	50	24	425.5→130.1	—5
Amine	HDA	Hexamethylenediamine	124-09-4	>99^a^	0.72	25	12	117.0→99.9	117.0→54.9
	2,6-TDA	2,6-Toluenediamine	823-40-5	>98^a^	1.30	35	16	122.9→105.9	1229→78.9
	IPDA^2^	Isophorone diamine	2855-13-2	>99^a^	1.72	30	14	171.1→154.0	171.1→80.9
	2,4-TDA	2,4-Toluenediamine	95-80-7	>98^a^	2.07	35	16	122.9→105.9	1229→78.9
	ANL	Aniline	62-53-3	>99^b^	2.41	30	16	93.8→76.8	93.8→50.9
	NDA	1,5-Diaminonaphthalene	2243-62-1	>97^a^	2.90	40	28	158.9→114.9	158.9→1426
	DPDA	4,4′-Diaminodiphenyl ether	101-80-4	>98^a^	3.94	45	22	200.9→107.9	200.9→79.9
	CHA	Cyclohexylamine	108-91-8	>99^a^	5.21	25	10	99.9→82.9	99.9→55.0
	4,4′-MDA	4,4′-Methylenedianiline	101-77-9	>98^a^	6.85	45	26	199.0→105.9	199.0→79.4
	H12MDA^4^	Dicyclohexylmethane-4,4′-diamine	1761-71-3	>97^a^	6.76, 6.95	30	18	211.1→80.9	211.1→95.0
	DABP	3,3′-Dimethyl-4,4′-methylenedianiline	838-88-0	>90^b^	7.89	50	28	227.0→120.0	227.0→76.9

1 Reagent purity supplied by (a) Tokyo Kasei Kogyo Co. Ltd., (b) Wako Pure Chemicals Co. Ltd.

2 Mixture of *cis-*isomer and *trans*-isomer.

3 Mixture of 2,2,4-TMDI and 2,4,4-TMDI.

4 Mixture of *cis, cis*-isomer, *cis*,*trans*-isomer and *trans*,*trans*-isomer.

5 Not detected.

Analytical grade methanol and acetonitrile were purchased from Merck (Darmstadt, Germany). Analytical grade acetone, acetic acid, formic acid, and dichloromethane were purchased from Sigma–Aldrich. Di-*n*-butylamine (DBA) was purchased from Tokyo Kasei Kogyo (Tokyo, Japan). Water was purified using the Milli-Q Gradient A10 system (Millipore, Billerica, MA).

### Analytical procedure for isocyanates

Samples were cut into small pieces. PUR foam samples (50 mg) or PUR-coated samples (10 cm^2^ of coated area) were placed in a 50 mL glass tube (24 mm i.d. × 220 mm) equipped with a screw cap, and 20 mL of DBA solution (0.1 mg/mL in dichloromethane) was added. After derivatization and extraction overnight at 60°C, the samples were removed and washed with 5 mL of dichloromethane. The solutions were combined and the dichloromethane was evaporated in a vacuum centrifuge (CVE-100; Tokyo Rikakikai, Tokyo, Japan). The residues (∼200 *μ*L of DBA) were then diluted to 1 mL with acetonitrile. The test solutions were subsequently evaluated using LC/MS/MS (Aquity TQD system; Waters, Milford, MA).

The LC/MS/MS analysis was performed with an Acquity Series LC–MS, Waters Corporation. The conditions were as follows: column: Acquity BEH C18 (100 × 2.1 mm, 1.7 *μ*m particle diameter); column temperature: 40°C; mobile phase: A, 0.1% formic acid; B, 0.1% formic acid/acetonitrile, A:B (40:60)—linear gradient (8 min)—A:B (5:95) (5 min); flow rate: 0.4 mL/min; injection volume: 1 *μ*L; method of ionization: electrospray ionization (+); capillary voltage: 3 kV; ion source temperature: 150°C; cone gas and flow rate: 50 L/h (nitrogen); desolvation temperature: 400°C; collision gas: 0.1 mL/min (argon); measurement mode: multiple-reaction monitoring (MRM). Cone voltages and collision energies for the MRM of each compound are shown in Table 1. Identification of the detected isocyanate was performed by confirming of the ion peak area ratio (quantitative/qualifying) and the MS/MS scan spectra.

### Analytical procedure for amines

The samples were cut into small pieces. PUR foam samples (50 mg) or PUR-coated samples (10 cm^2^ of coated area) were placed in a 50 mL glass tube equipped with a screw cap, and 20 mL of methanol was added. After extraction overnight at 60°C, the samples were removed and washed with 5 mL of methanol. The solutions were combined, and 1 mL of 4% acetic acid was added. The solution was evaporated to a volume of ∼1 mL, and then diluted to 10 mL with 4% acetic acid. The test solutions were subsequently evaluated by LC/MS/MS.

The LC/MS/MS conditions were as follows: column: Acquity HSS T3 (100 × 2.1 mm, 1.7 *μ*m particle diameter); mobile phase: A, 0.1% formic acid; B, 0.1% formic acid/methanol, A:B (99:1)—linear gradient (5 min)—A:B (5:95) (2 min); flow rate: 0.3 mL/min. Cone voltages and collision energies for the MRM of each compound are shown in Table 1. All other conditions were the same as for the isocyanate analysis. Identification of the detected amine was performed by confirmation of the ion peak area ratio (quantitative/qualifying) and the MS/MS scan spectra.

### Preparation of calibration solutions for LC/MS/MS

The stock solution of isocyanate was diluted to concentrations of 10, 25, 50, 100, 250, 500, and 1000 ng/mL with dichloromethane. Twenty milliliters of DBA solution was spiked with 100 *μ*L of each standard solution and allowed to sit overnight at 60°C. The dichloromethane was then evaporated in a vacuum centrifuge. The residues were diluted to 1 mL with acetonitrile to give final concentrations of 1–100 ng/mL. The stock solution of amine was diluted with 4% acetic acid to give final concentrations of 0.2–50 ng/mL.

## Results

### Analytical method for isocyanates

Fourteen aliphatic and aromatic isocyanates are listed in Regulation (EU) No 10/2011. Of these, diphenylmethane-2,4′-diisocyanate (2,4′-MDI) and 2,4-TDI dimer cannot be used as standard reagents. In addition, 4,4′-oxybis(diphenyldiisocyanate), 1,5-diisocyanatonaphthalene, and 4,4′-diisocyanato-3,3′-dimethylbiphenyl standards are not soluble in toluene or dichloromethane; thus, they were not analyzed. Phenyl isocyanate (PI), a base compound of aromatic isocyanates, was added, producing 10 different isocyanates for quantification.

Many isocyanate analysis methods have been previously reported. In these methods, the use of DBA derivatization is advantageous in its high reactivity, and the fact that it allows for extremely sensitive and selective LC/MS/MS detection. The conditions for the derivatization were optimized for quantification of the residual isocyanate in PUR products according to the literature (Marand et al. 2004; Karlsson et al. 2005).

When performing electrospray ionization (ESI)–MS of the isocyanate–DBA derivatives, the most abundant ions were [M+H]^+^ (M is the molecular weight of isocyanate derivative). Performing MS/MS product ion scan of the [M+H]^+^ of the isocyanate–DBA derivatives, the [DBA+H]^+^ (*m/z* 130) ions were all present as the most abundant ion. Therefore, [DBA+H]^+^ ions from [M+H]^+^ were used as quantitative ion. The [M+H-DBA]^+^ (*m/z* = M + 1–129) ions were used as qualifying ions for identification (Table 1).

**Figure 1:**
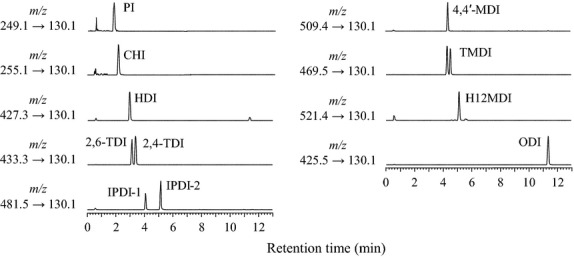
LC/MS/MS chromatograms of isocyanate solutions (10 ng/mL). Data acquisition was performed in the MRM mode using the ions listed in Table 1. LC/MS/MS, liquid chromatography–tandem mass spectrometry; MRM, multiple-reaction monitoring.

LC separation was performed with a gradient mobile phase using water and acetonitrile with 0.1% formic acid. Most isocyanate–DBA derivatives were separated. The chromatograms of the standard solutions are shown in Figure 1. Since the isophorone diisocyanate (IPDI) and trimethylhexamethylene-1,6-diisocyanate (TMDI) standard reagents used in this study were mixtures of isomers, they were detected as two peaks. As the two peaks of TMDI had similar retention times, the quantification was performed by summing the areas of the peaks. On the other hand, the two peaks from the IPDI were quantified individually by using a ratio of the areas of the two peaks. Although the dicyclohexylmethane-4,4′-diisocyanate (H12MDI) reagent was present as a mixture of four isomers, this was detected as a single peak.

Dichloromethane was used for extraction of isocyanate in consideration of the solubility and stability of the compound. Preliminary analysis of kitchen sponge 1 showed the presence of 2,4-and 2,6-TDI; therefore, the derivatization and extraction conditions were optimized using this sample. Fifty milligrams of sample was immersed in 0.001–0.2 mg/mL of DBA solution, and left for 6, 12, 18, or 24 h at 60°C. Test solutions were then prepared from each extract, and the peak areas of the 2,4-TDI and 2,6-TDI derivatives were compared. The peak areas remained constant between 12 and 24 h of extraction at DBA concentrations of 0.05–0.2 mg/mL. However, when the 0.2 mg/mL DBA solution was used, unreacted DBA interfered with the separation of PI and cyclohexyl isocyanate (CHI). Therefore, the extraction and derivatization of isocyanates were performed for 18 h with a 0.1 mg/mL DBA solution.

### Analytical method for amines

Measurements were attempted for the 13 amines obtained from the isocyanates listed in Regulation (EU) No 10/2011, in addition to PI. However, trimethylhexamethylene-1,6-diamine could not be obtained as a standard reagent, and octadecylamine was not soluble in 4% acetic acid. Therefore, 11 different amines were quantified.

LC/MS/MS detection without derivatization has been previously reported for amine analysis (Mortensen et al. 2005). When performing the ESI–MS, the typical ions were found to be [M+H]^+^, and the main product ions from [M+H]^+^ were used for identification, as shown in Table 1.

**Figure 2:**
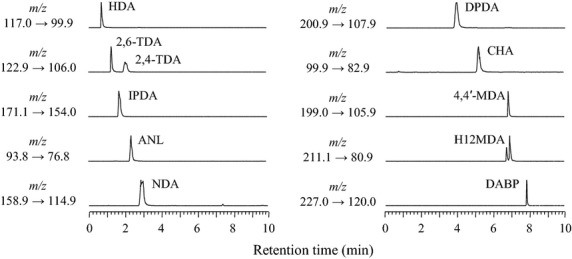
LC/MS/MS chromatograms of amine standard solutions (10 ng/mL). Data acquisition was performed in the MRM mode using the ions listed in Table 1. LC/MS/MS, liquid chromatography–tandem mass spectrometry; MRM, multiple-reaction monitoring.

LC separation was performed with a gradient mobile phase using water and methanol with 0.1% formic acid on an Acquity HSS T3 column for polar compound retention. As the H12MDA had two isomer peaks, quantification was performed by summing the two peak areas. The chromatograms of the standard solutions are shown in Figure 2.

Methanol was used for extraction of amines in consideration of the solubility of the compound. Moreover, methanol would react with the free isocyanates and prevent them from hydrolyzing to amines. The eight different isocyanates, hexamethylene diisocyanate (HDI), 2,4-and 2,6-TDI, IPDI, PI, CHI, 4,4′-MDI, and H12MDI (2 *μ*g each) were spiked into 20 mL of methanol for the test procedure. The corresponding amines were not detected in significant quantities (<1% of spiked amount). H12MDA was detected at a level of 14% in spiked H12MDI; therefore, the H12MDA was presumed to include some hydrolyzed H12MDI when carrying out the quantification.

The extraction conditions were optimized by using kitchen sponge 1, which contained 2,4-and 2,6-TDA. As the extracted amine levels plateaued after 12 h, the extraction time was selected as 18 h in methanol in order to ensure maximum recovery of the compounds.

### Recovery tests

When 100 *μ*L of isocyanate solution (each 100 ng/mL) was spiked directly into the sample (50 mg of kitchen sponge 1 or 10 cm^2^ of glove 1), their recoveries were poor. Although the multiple extraction steps were tried, there was no improvement effect of residual levels and recoveries. It was speculated that the isocyanates reacted with functional groups present on the sponge/glove polymer, or that they decomposed to amines. Therefore, in this study, the isocyanates were spiked immediately after immersing the sample in the extraction solution.

Isocyanates were spiked at 10 ng (0.2 mg/kg or 1 ng/cm^2^) based on the residual levels in samples. Recoveries of 2,4-and 2,6-TDI, and 4,4′-MDI, which were used as the main monomers, were found to be in the range of 90–124% (Table 2). The standard deviations for these monomers were high because the samples themselves contained them. Since isocyanates are unstable compounds, possibly the residual levels in material varied with the sample piece. In this study, since it was apprehensive about dichloromethane affecting the material, the method for removing the background by pre-extraction before spiking was not considered. On the other hand, recoveries of HDI, IPDI-1, PI, CHI, and ODI from glove 1 were <10–65%. It was speculated that the isocyanates added to glove 1 were adsorbed onto the nylon fibers. These recoveries had not improved by multiple extraction steps. Therefore, the residual levels of these compounds were quantified as reference value.

**Table 2:** Recoveries and quantification limits of isocyanates and amines.

Group	Compound	Recovery, %		Limit of quantification	
		Kitchen sponge 1	Glove 1	mg/kg	ng/cm^2^
Isocyanate	PI	112 *±* 7	53 ± 5	0.1	0.5
	CHI	85 *±* 4	39 ± 4	0.1	0.5
	HDI	78 *±* 12	65 ± 2	0.02	0.1
	2,6-TDI	111 *±* 32	113 ± 9	0.02	0.1
	2,4-TDI	94 *±* 20	124 ± 9	0.02	0.1
	IPDI-1	47 *±* 3	63 ± 4	0.04	0.2
	IPDI-2	104 *±* 7	87 ± 2	0.04	0.2
	TMDI	78 *±* 5	82 ± 4	0.02	0.1
	4,4′-MDI	90 *±* 14	101 ± 16	0.02	0.1
	H12MDI	111 *±* 7	105 ± 2	0.02	0.1
	ODI	60 *±* 3	<10	0.02	–
Amine	HDA	30 *±* 8	47 ± 14	–	–
	2,6-TDA	88 *±* 2	66 ± 4	1	50
	IPDA	99 *±* 3	83 ± 3	0.2	50
	2,4-TDA	89 *±* 5	67 ± 2	1	50
	ANL	98 *±* 0	90 ± 3	0.2	1
	NDA	100 *±* 6	98 ± 8	1	5
	DPDA	88 *±* 5	91 ± 3	0.2	1
	CHA	90 *±* 2	91 ± 2	0.2	1
	4,4-MDA	88 *±* 5	93 ± 1	0.2	5
	H12MDA	89 *±* 4	84 ± 4	1	5
	DABP	93 *±* 5	86 ± 4	0.2	1

Each value is the mean ± SD of three trials. Isocyanates were spiked 10 ng in sponge 1 (50 mg) or glove 1 (10 cm^2^). Amines were spiked 500 ng in sponge 1 (50 mg) or glove 1 (10 cm^2^).

Evaluation of the recovery of the amines was carried out by directly spiking into kitchen sponge 1 (50 mg) and glove 1 (10 cm^2^), as shown in Table 2. Amines were spiked at 500 ng (10 mg/kg or 50 ng/cm^2^) based on the residual levels in the samples. Recoveries of 2,4-and 2,6-TDA, and 4,4′-MDA, which were the main residual amines, were 66–93%, with standard deviations of 1–5%. The recoveries of 2,4-and 2,6-TDA from the glove sample were quite low. Recoveries of HDA were not sufficient for quantification to be carried out. Other amines were found at levels of 83–100%, with standard deviations of 0–8%. In the case of 50 ng spiking, recoveries of HDA, 2,4-and 2,6-TDA, and IPDA from glove 1 were <50%.

The limits of quantification (LOQ) defined as signal to noise ratio of 10. However, the recoveries of 2,6-TDA, IPDA, and 2,4-TDA for glove 1 were not enough (less than 30%) by the recovery test spiked at 5 ng/cm^2^. From these results, the LOQ of the isocyanates determined to 0.02–0.1 mg/kg or 0.1–0.5 ng/cm^2^, those of the amines (excluding HDA) determined to 0.2–1 mg/kg or 1–50 ng/cm^2^.

### Residual levels in PUR foam products

The residual levels of isocyanates and amines in three cushionings for fruits and 12 samples of kitchen sponge are quantified. Although kitchen sponges are not food contact material, since their materials were similar to cushionings for fruits. Therefore, the residual levels in kitchen sponges were compared with the regulation value as reference of cushionings. The results are shown in Tables 3, 4.

**Table 3:** Residual levels of isocyanates in flexible polyurethane foam products.

Sample	No.	Residual level, mg/kg				
		2,6-TDI	2,4-TDI	4,4′-MDI	Total	Total (as NCO)
Cushioning for fruits	1	0.25	0.13	ND	0.38	0.19
	2	0.11	0.09	ND	0.20	0.10
	3	0.03	0.07	ND	0.10	0.05
Kitchen sponge	1	0.23	0.92	ND	1.15	0.55
	2	0.26	0.68	ND	0.94	0.46
	3	ND	0.04	ND	0.04	0.02
	4	0.02	0.28	0.30	0.60	0.25
	5	0.20	0.56	ND	0.77	0.37
	6	0.04	0.10	ND	0.14	0.07
	7	0.05	0.11	ND	0.16	0.08
	8	ND	0.07	ND	0.07	0.03
	9	ND	0.05	ND	0.05	0.02
	10	ND	ND	0.03	0.03	0.01
	11	0.13	0.11	ND	0.24	0.12
	12	ND	ND	0.15	0.15	0.05

Each value is the mean of three trials. ND: <quantification limit. Isocyanates that are not listed were ND for all samples.

**Table 4:** Residual levels of amines in flexible polyurethane foam products.

Sample	No.	Residual level, mg/kg				
		2,6-TDA	2,4-TDA	ANL	CHA	Total
Cushioning for fruits	1	26	59	1.3	1.4	89
	2	25	57	0.8	ND	83
	3	10	19	ND	ND	29
Kitchen sponge	1	8.5	17	ND	ND	26
	2	11	21	ND	ND	33
	3	11	23	ND	ND	35
	4	11	25	ND	ND	36
	5	12	22	ND	ND	35
	6	12	23	ND	ND	35
	7	13	24	ND	ND	38
	8	8.2	17	ND	ND	26
	9	11	21	ND	ND	32
	10	4.0	10	ND	ND	14
	11	10	22	ND	ND	32
	12	7.0	15	ND	ND	23

Each value is the mean of three trials. ND: <quantification limit. Amines that are not listed were ND for all samples.

Levels of 2,4-TDI and 2,6-TDI were found to be 0.04–0.92 and 0.02–0.26 mg/kg, respectively, in most samples. In addition, 4,4′-MDI, which is used as an adhesive to bond different parts of the sponges, was detected in three kitchen sponges at levels of 0.03–0.30 mg/kg. PI and CHI were detected in cushioning 1 at levels below the LOQ, and were deduced to be present as impurities or decomposition products of 2,4-and 2,6-TDI. No other types of isocyanate were detected. The total amount of isocyanates was found to be 0.03–1.15 mg/kg (0.01–0.55 mg/kg as NCO). No samples exceeded 1.0 mg/kg (as NCO), which is the maximum level specified in Regulation (EU) No 10/2011 for food contact materials. It should be noted that kitchen sponges are not classified as food contact materials.

Levels of 2,4-TDA and 2,6-TDA were quantified to be 10–59 and 7.0–26 mg/kg, respectively, in all samples. In addition, small amounts of aniline (ANL) or cyclohexylamine (CHA) were detected in cushionings 1 and 2. No other types of amines were detected. The total amount of amines was 14–89 mg/kg.

Commercial flexible PUR foam is manufactured using 2,4-and 2,6-TDI as raw materials. Residual amine levels were 18–633 times higher than isocyanate levels, with no correlation observed between them. This was attributed to the isocyanates that did not react during the polymerization process remaining as amines in the PUR foam products. On the other hand, a significant correlation was observed between 2,4-TDA and 2,6-TDA levels (*R*^2^ = 0.976). Flexible PUR foam is usually manufactured from a mixture of 2,4-and 2,6-TDI (Takayanagi 2000). The ratio of residual amounts of 2,4-TDA/2,6-TDA was calculated to be 1.8–2.5.

### Residual levels in PUR-coated products

The residual levels of isocyanates and amines were quantified in the PUR-coated section of two types of gloves (glove 1: 23.4 mg/cm^2^, glove 2: 37.0 mg/cm^2^), and one pastry bag (119 mg/cm^2^). The levels of the isocyanates and amines detected in the samples are shown in Table 5.

**Table 5:** Residual levels of isocyanates and amines in polyurethane-coated products.

Sample	Residual level, mg/kg									*μ*g/product
	2,6-TDI	IPDI-1	IPDI-2	2,4-TDI	PI	CHI	4,4′-MDI	H12MDI	Total	Total
Glove 1	0.013	ND	ND	ND	ND	ND	0.064	ND	0.077	0.40
Glove 2	ND	ND	0.011	ND	ND	ND	3.0	0.022	3.0	18.7
Pastry bag	0.029	ND	ND	0.004	ND	ND	0.014	ND	0.047	7.4
	2,6-TDA	IPDA		2,4-TDA	ANL	CHA	4,4′-MDA	H12MDA	Total	Total
Glove 1	ND	ND		ND	1.0	ND	29	ND	30	106
Glove 2	ND	ND		ND	0.73	0.43	0.92	3.5	5.6	35
Pastry bag	ND	ND		ND	ND	ND	ND	ND	ND	ND

Each value is the mean of three trials. ND: <quantification limit. Isocyanates and amines that are not listed were ND for all samples.

4,4′-MDI was identified in all samples, with a particularly high level of 3.0 mg/kg found in glove 2. These PUR-coated gloves were manufactured using 4,4′-MDI as the main raw material. 2,4-TDI was found in the pastry bag, while 2,6-TDI was identified in the pastry bag and glove 1. In addition, IPDI and H12MDI impurities or decomposition products of the isocyanates used as raw materials were detected. No other types of isocyanates were detected. The total amounts of isocyanates found in glove 1, glove 2, and the pastry bag were 0.077, 3.0, and 0.047 mg/kg, respectively (0.028, 1.0, and 0.021 mg/kg as NCO). The residual levels in the product were 0.4, 18.7, and 7.4 *μ*g/product, respectively, with the level for the pastry bag found to be half that of glove 2.

ANL, CHA, 4,4′-MDA, and H12MDA were all identified in both glove types, with total amine quantities of 30 and 5.6 mg/kg, respectively. The major amine contribution in glove 1 was 4,4′-MDA, while that in glove 2 was found to be H12MDA. It was speculated that H12MDI was used for the surface treatment when producing glove 2. In contrast, no amines were detected in the pastry bag. When the residual levels of amines and isocyanates in the two gloves were compared, no correlation was observed, the same as in the case of the PUR foam samples.

## Discussion

An analytical method for identifying 10 different isocyanates and 11 different amines in PUR foam and PUR-coated products using LC/MS/MS was developed and optimized. Since isocyanates decompose to amine easily, isocyanates do not exist in the product surface. The residual isocyanates existed in the inside of the PUR material. In this study, we quantify the extractable isocyanate with dichloromethane. The recoveries of certain isocyanates and amines were low. Although we tried the improvement of these recoveries, they have not improved. Therefore, the residual levels of these compounds were quantified as reference value. Furthermore, the identification of suitable internal standards for the isocyanates recovered at unsatisfactory levels would greatly improve the accuracy of the methodology. But, the main compounds used as monomers in the production of PUR products, and their decomposition species, were clearly identified at quantifiable levels.

The residual levels of isocyanates and amines in commercial PUR products were quantified using the LC/MS/MS technique. Commercial PUR foam is manufactured using 2,4-and 2,6-TDI as raw materials; however, these were only detected at low levels. On the other hand, 2,4-and 2,6-TDA, which are hydrolysates of 2,4-and 2,6-TDI, were detected at levels 18–633 times that of the corresponding isocyanates. PUR-coated gloves are manufactured using 4,4′-MDI as the main raw material, and a large amount of this compound, in addition to 4,4′-MDA and H12MDA, remained in these samples. 2,4-and 2,6-TDI, and 4,4′-MDI were detected in the pastry bag; however, no amines were detected.

In this study, the residual levels in kitchen sponges were quantified as reference of cushionings. As a result, there were no samples that exceeded the level of 1.0 mg/kg (as NCO) as specified in Regulation (EU) No 10/2011. Since PUR foam and PUR-coated gloves are mainly used for dry foods, it is thought that there is almost no release of amines. However, as absorbent pads for fish and meats contain similar levels of amine to kitchen sponges, caution should be applied when using these in case of migration of the aromatic compounds 2,4-and 2,6-TDA, and 4,4′-MDA, in particular.
